# Indoor Air Quality Prior to and Following School Building Renovation in a Mid-Atlantic School District

**DOI:** 10.3390/ijerph182212149

**Published:** 2021-11-19

**Authors:** Sandra E. Zaeh, Kirsten Koehler, Michelle N. Eakin, Christopher Wohn, Ike Diibor, Thomas Eckmann, Tianshi David Wu, Dorothy Clemons-Erby, Christine E. Gummerson, Timothy Green, Megan Wood, Ehsan Majd, Marc L. Stein, Ana Rule, Meghan F. Davis, Meredith C. McCormack

**Affiliations:** 1 Division of Pulmonary, Critical Care, and Sleep Medicine, Yale University School of Medicine, New Haven, CT 06510, USA; sandra.zaeh@yale.edu; 2 Division of Pulmonary and Critical Care Medicine, Johns Hopkins School of Medicine, Baltimore, MD 21287, USA; meakin1@jhmi.edu (M.N.E.); teckman1@jhmi.edu (T.E.); 3 Department of Environmental Health and Engineering, Johns Hopkins Bloomberg School of Public Health, Baltimore, MD 21287, USA; kkoehle1@jhu.edu (K.K.); dclemo10@jhu.edu (D.C.-E.); tgreen3@jhmi.edu (T.G.); mwood39@jhu.edu (M.W.); arule1@jhu.edu (A.R.); mdavis65@jhu.edu (M.F.D.); 4 Baltimore City Public Schools, Baltimore, MD 21287, USA; crwohn@bcps.k12.md.us (C.W.); idiibor@bcps.k12.md.us (I.D.); 5 Division of Pulmonary, Critical Care, and Sleep Medicine, Baylor College of Medicine, Houston, TX 77030, USA; david.wu@bcm.edu; 6 Center for Innovations in Quality, Effectiveness, and Safety, Michael E. DeBakey VA Medical Center, Houston, TX 77030, USA; 7 Division of Neurology, Yale University School of Medicine, New Haven, CT 06510, USA; christine.gummerson@yale.edu; 8 Division of Mobile Source Control, California Air Resources Board, Sacramento, CA 95817, USA; ehsan.majd@arb.ca.gov; 9 Johns Hopkins School of Education, Baltimore, MD 21287, USA; m.stein@jhu.edu; 10 Baltimore Education Research Consortium, Baltimore, MD 21287, USA; 11 Department of Molecular and Comparative Pathobiology, Johns Hopkins School of Medicine, Baltimore, MD 21287, USA

**Keywords:** schools, indoor air quality, renovation

## Abstract

Children spend the majority of their time indoors, and a substantial portion of this time in the school environment. Air pollution has been shown to adversely impact lung development and has effects that extend beyond respiratory health. The goal of this study was to evaluate the indoor environment in public schools in the context of an ongoing urban renovation program to investigate the impact of school building renovation and replacement on indoor air quality. Indoor air quality (CO_2_, PM_2.5_, CO, and temperature) was assessed for two weeks during fall, winter, and spring seasons in 29 urban public schools between December 2015 and March 2020. Seven schools had pre- and post-renovation data available. Linear mixed models were used to examine changes in air quality outcomes by renovation status in the seven schools with pre- and post-renovation data. Prior to renovation, indoor CO measurements were within World Health Organization (WHO) guidelines, and indoor PM_2.5_ measurements rarely exceeded them. Within the seven schools with pre- and post-renovation data, over 30% of indoor CO_2_ measurements and over 50% of indoor temperatures exceeded recommended guidelines from the American Society of Heating, Refrigerating, and Air Conditioning Engineers. Following renovation, 10% of indoor CO_2_ measurements and 28% of indoor temperatures fell outside of the recommended ranges. Linear mixed models showed significant improvement in CO_2_, indoor PM_2.5_, and CO following school renovation. Even among schools that generally met recommendations on key guidelines, school renovation improved the indoor air quality. Our findings suggest that school renovation may benefit communities of children, particularly those in low-income areas with aging school infrastructure, through improvements in the indoor environment.

## 1. Introduction

Americans spend about 90% of their time indoors, and for children, a substantial portion of this time is spent in the school setting [1]. Indoor air quality is particularly relevant for children. Air pollution has been shown to impact lung growth and contribute to the risk of asthma exacerbation [2], a leading reason for school absences. Studies have linked air quality and thermal comfort to student performance [3,4,5].

The indoor environment is modifiable and studies have focused on interventions in the home setting as a means to improve the health of children and other household members [6,7,8]. Interventions in the school environment provide a means to positively impact children at a community level and to benefit many children by interventions focused on shared, common spaces.

As school infrastructure in the United States ages, school buildings require increased maintenance, and school environmental conditions may be less conducive to optimal health and learning. In 2017, nationwide infrastructure reports showed that almost a quarter of public schools with permanent buildings in the United States were rated as being in “fair” or “poor” condition [9]. There are significant disparities in funding for improvement and maintenance of facilities, with prior evidence showing that capital funds for school facility improvement often fail to reach schools with children from low income communities [10].

Prior research from our group has shown that worse school infrastructure is associated with worse student achievement [11]. Additionally, building characteristics within schools such as physical defects (i.e., leaks in roof, broken windows, damaged walls) are associated with increased indoor air pollution (higher indoor NO_2_ and indoor CO levels) [12]. Poor air quality can also be a concern during the process of school renovation due to the release of toxic materials during demolition, dust and fumes from construction, and designs that interfere with ventilation [13]. Few studies have directly assessed school environmental conditions and very few have measured the impact of renovation or improvement of school facilities after renovation has been completed [14]. An ongoing school renovation program in an urban environment within the mid-Atlantic region (including the states of Delaware, Maryland, New Jersey, and Pennsylvania) provided the opportunity to apply a natural experimental study design to assess the impact of renovation on indoor air quality. The urban renovation program was launched in 2010 with the aim of addressing the city’s aging school buildings and infrastructure [15].

Our aim was to evaluate environmental conditions in schools that were included in the urban renovation program to investigate the impact of school building renovation and replacement on indoor air quality. We hypothesized that school building renovation would lead to improved indoor air quality. We performed a detailed assessment of indoor air quality measures that have been shown to have an impact on health and performance (including CO_2_, PM_2.5_, CO, and temperature) [16,17,18,19,20].

## 2. Methods

### 2.1. School Characterization

To guide prioritization of schools for renovation, a districtwide facility condition assessment was initially performed by an independent engineering firm (Jacobs Engineering Group, Dallas, TX, USA) [21]. The facility condition index (FCI), a national standard for the overall condition of a building, was calculated for every school [22]. The FCI is derived as a ratio of the cost to repair a given school to the cost of constructing a theoretical school with similar characteristics. FCI ≤ 10% is good condition; 11–30% is average; 31–50% is poor; 51–74% is very poor; ≥75% is a candidate for replacement [21].

Twenty-nine schools in the ongoing school renovation program were enrolled between December 2015 and March 2020. Institutional review board (IRB) approvals were obtained from the Johns Hopkins School of Public Health IRB and the IRB of the participating school district. Three schools entered the study after renovation had been completed and contributed only post-renovation data, seven schools contributed data pre- and post-renovation, and the remaining nineteen schools contributed only pre-renovation data. Within this study, our analysis focuses on the seven schools with both pre- and post-renovation data. Air quality measurements were stopped in March of 2020 due to the COVID-19 pandemic.

### 2.2. Renovation

The renovations performed on each school within the urban renovation program were heterogeneous and tailored to the needs of the individual school. Of the eleven schools in which post-renovation data was assessed, nine schools had additions and substantial renovations performed (including new heating, ventilation, and air conditioning, or HVAC systems, and window replacement) and two schools were replaced [23]. For replacement schools, entirely new school buildings were constructed. One school was constructed at the same site and the other replacement school was constructed behind the existing school. Within the seven schools with pre- and post-renovation data, six schools had renovations performed and one school was replaced. All seven schools had new HVAC systems installed.

### 2.3. Procedures for Air Quality Monitoring within Schools

Within each school, indoor air quality was assessed at three time points within a given academic year. Over 1400 school days of monitoring were performed throughout the study. For most schools, this included a visit in the fall (September to November), winter (December to February), and spring (March to May). Within each season school visit, two weeks of monitoring was performed, with five consecutive days (Monday-Friday) in each week (mean indoor visit length: 89.7 ± 16.7 h). In the first week, one classroom and one common area were monitored, and in the second week, a different classroom and common space were monitored. Common spaces included areas such as the gymnasium, cafeteria, or library. Environmental equipment was placed on a portable table provided by the study team in a protective wire cage/frame with signs that explained the purpose of the equipment. CO_2,_ indoor PM_2.5_, CO, and indoor temperature were measured at the same location.

Within monitoring locations, particulate matter with an aerodynamic diameter smaller than 2.5 μm (PM_2.5_) was measured using a Personal DataRam (pDR) monitor, model pDR-1200 (Thermo Electron, Franklin, MA, USA), with 0.001–400 mg/m^3^ concentration measurement range and a 5-min logging resolution. The pDR was connected to a BGI model 400 s personal sampling pump (BGI INC., Waltham, MA, USA) operating at the air flow of 4 L/min, and a BGI model GK 2.05 (Mesa Labs, Butler, NJ, USA) inlet cyclone with a 50% cut point of 2.5 μm. Filter samples collected downstream of the pDR nephelometer were collected on 37-mm, 2.0 μm pore-size PTFE membrane filters and were used to correct concentrations from the pDR for gravimetric time-weighted average (TWA) concentrations. pDR concentrations were also humidity corrected using standard approaches [24]. Filters were preconditioned for 24 h in a humidity and temperature controlled room prior to being weighed in the same room. Indoor PM samples included at least 10% blanks and duplicates and all reported concentrations were blank corrected.

CO_2_ and CO were measured at 1-min intervals using a direct-reading Advanced Sense Pro indoor air quality (IAQ) meter (GrayWolf Sensing Solutions LLC, Shelton, CT, USA). A dual-wave NDIR IR-Infrared sensor was used to measure CO_2_ while an electromechanical sensor was used to detect CO. The IAQ meter was factory calibrated annually and checked for quality control weekly using a calibration kit provided by the manufacturer for a one point calibration with zero air starting in April 2018. Prior to that only annual calibrations were used. Within each monitoring location, the IAQ meter was placed on a table approximately 1 m away from the ground and away from air vents, windows, and doors, as possible. Indoor temperature measurements were obtained using HOBO data loggers (HOBO U12 Data Logger, Onset Computer Corporation, Bourne MA, USA).

Outside of each school, samplers were placed on the roof to assess the contribution of outdoor air to indoor air quality. Outdoor sampling at each school occurred throughout both weeks of indoor air quality monitoring (including the weekend between the two indoor sampling weeks) (mean outdoor visit length: 240.5 ± 48.9 h). A SKC Personal Environmental Monitor (PEM) for PM_2.5_ with the same type of filter used for the pDR was connected to an Allegro model 9805 diaphragm sampling pump (Allegro Industries, Piedmont, SC, USA), operating at the air flow of 4 L/min. A battery powered iButton data logger (iButtonLink, Whitewater, WI, USA) was used to measure outdoor temperature at 10-min intervals.

### 2.4. Statistical Analysis

For each monitored location, CO_2_ and CO were monitored at 1-min resolution and indoor PM_2.5_ was monitored at 5-min resolution. For indoor temperature, data were first measured at 10-min intervals and then averaged at the hour level, taking the mean of each hour’s six measurements. School day mean values (8 AM to 4 PM) of CO_2_, PM_2.5_, CO, and indoor temperature were calculated. During data quality control assessments, CO_2_ data was filtered to remove values >4000 ppm and <250 ppm as a school day mean (3.9% of days). The limit of detection was 1 µg/m^3^ for the PM_2.5_; for school day mean values below the LOD, a value of LOD/√2 (0.7 µg/m^3^) was imputed (4.6% of days). CO data was reported at intervals of 1 ppm and was filtered to remove values for which 0 ppm was reported for more than 90% of the school day (27.3% of days).

For each monitored location, outdoor PM_2.5_ and temperature were monitored. There was one outdoor sample collected for PM_2.5_ over the two week period per school, resulting in one value for approximately 12 days of sampling. Outdoor temperature data was measured at 10-min intervals and then averaged at the hour level, taking the mean of each hour’s six measurements. School day mean values (8 AM to 4 PM) of outdoor temperature were calculated.

Descriptive statistics (median and interquartile range, IQR) were calculated for all air quality measures prior to and post-renovation. CO_2_ was compared to an indoor concentration of 1000 ppm as recommended by the American Society of Heating, Refrigerating, and Air Conditioning Engineers (ASHRAE) [25]. PM_2.5_ and CO values were compared to corresponding indoor WHO guidelines (25 µg/m^3^ for PM_2.5_ and 7 mg/m^3^ [~6.1 ppm at 25 °C for CO] as a 24 h average) [26].

For indoor temperature, in addition to describing the average school day value, the proportion of respective temperature measurements taken at 10 min resolution at each site during the school day that were outside of the recommended value/range based on standards from ASHRAE were calculated prior to and following renovation. For indoor temperature, recommended seasonal temperatures from ASHRAE assume slow air moment and 40% indoor relative humidity. The recommended indoor temperature ranges are 68–75 °F or 20–23.9 °C in fall/winter and 73–80 °F or 22.8–26.7 °C in spring/summer [27,28]. Temperature measurements were designated as being in range if they were within recommended ASHRAE guidelines based on season of assessment. Temperatures were considered to be too cold or too warm if they were below or above the limit of the ASHRAE recommended range for the season by 4 °C or less. Extreme values were defined as values that were more than 4 °C outside of the ASHRAE recommended range (too warm or too cold). The sum of temperature measurements that were assigned as extremely too cold, too cold, too warm, and extremely too warm divided by the total number of temperatures measurements were determined to be the proportion out of range for each school, location, and season.

Within the seven schools with pre- and post-renovation data, we assessed four indoor environmental parameters (CO_2_, PM_2.5_, CO, and proportion of temperature out of range) and two outdoor environmental parameters (PM_2.5_ and average school daily temperature) using six separate linear mixed models. Given that we had multiple time points of data measurement nested within each school, and to account for missing data, we used maximum likelihood estimates. Linear mixed models were used to account for intra-cluster variance of observations within schools. A mean school daily value of CO_2_, indoor PM_2.5_, CO, and a total proportion of temperature out of range from each of the four locations within each school during each visit was included in the model. A mean school daily value of outdoor temperature and a two-week value of outdoor PM_2.5_ within one location outside of each school was included in the model. CO_2_, indoor and outdoor PM_2.5_, and CO school daily mean values were log-transformed. Each model was controlled for renovation status of the school and season (fall, winter, spring). The results of a similar analysis incorporating all schools within our sample are included in the Online Supplement.

## 3. Results

Prior to renovation, IAQ measures (CO_2_, PM_2.5_, and CO) and temperature were measured in 26 schools and post-renovation IAQ measures and temperature were measured in 11 schools. Schools had a median Facility Condition Index (FCI) of 70.6 (IQR: 59.3–91.75), which corresponded to the very poor category. The majority of students were non-white (median 98.8%; IQR: 92.7–99.1%) with 93.7% (IQR: 89.5–96.1%) of free and reduced lunch meals (FARMS) provided in the 2014–2015 school year. The median year of school construction was 1958, with the oldest school constructed in 1910 and the most recent in 1981. Within the seven schools that had pre- and post-renovation data, the median FCI was 76.4 (IQR: 65.1–96.5), and the median year of construction was 1944 (Table 1).

### 3.1. Indoor Air Quality Measures

Within the seven schools that had pre- and post-renovation data, the median average school daily CO_2_ was 774.0 ppm (IQR: 626.3–1073.2 ppm), with over 30% of average school daily CO_2_ measurements exceeding ASHRAE guidelines. Following renovation, the median average school daily CO_2_ was 542.4 ppm (IQR: 460.3–679.4 ppm), with over 10% of average school daily CO_2_ measurements exceeding ASHRAE guidelines (Table 2). Prior to renovation, the median average school daily PM_2.5_ was 5.9 µg/m^3^ (IQR: 3.8–9.5 µg/m^3^) with average school daily PM_2.5_ exceeding WHO guidelines 2% of the time. Following renovation, the median average school daily PM_2.5_ was 2.7 µg/m^3^ (IQR: 1.4–5.2 µg/m^3^) with all average school daily PM_2.5_ measurements below WHO guidelines. Prior to renovation, the median school daily CO was 0.4 ppm (IQR: 0.3–0.6 ppm) and all values were within indoor air quality guidelines from the WHO. Following renovation, median average school daily CO was 0.3 ppm (IQR: 0.1–0.6 ppm). Indoor air quality metrics within the overall sample of 29 schools were similar to the seven schools with pre- and post-renovation data and are shown in Online Supplement Table S1.

**Figure 1:**
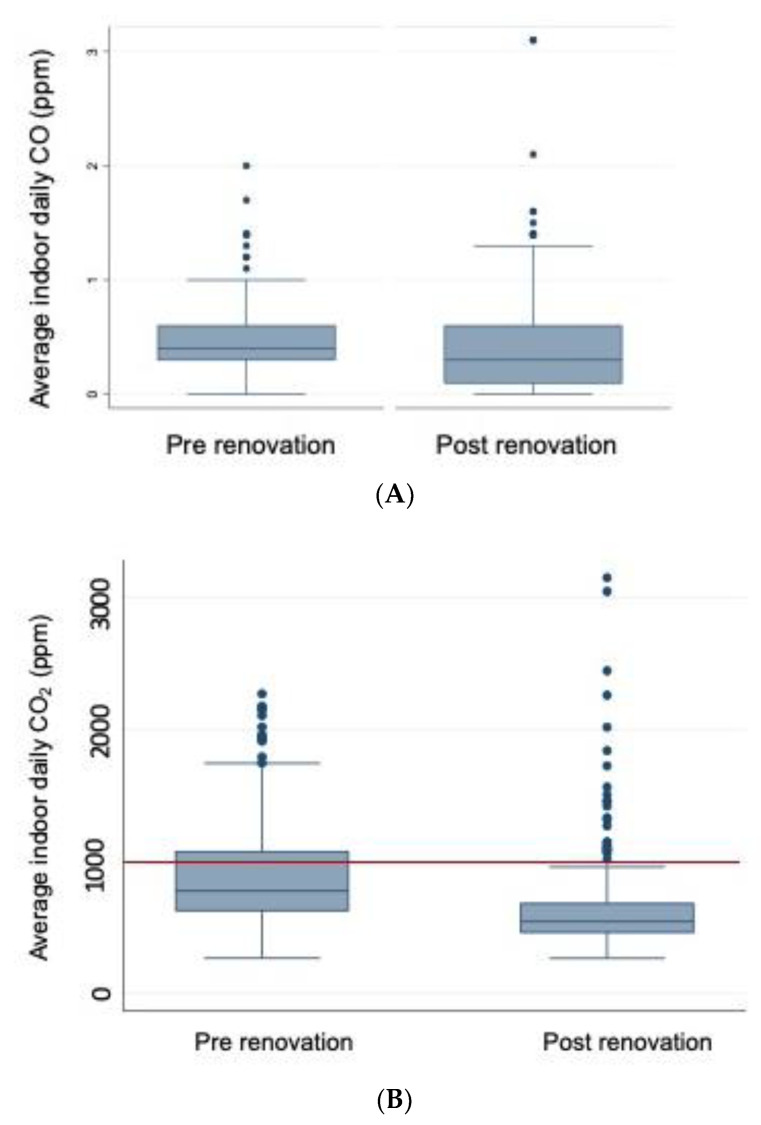

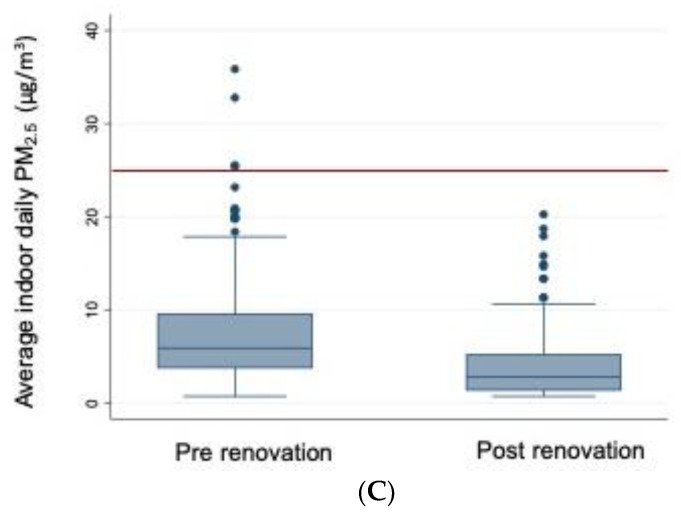
Indoor air quality measures by school renovation status in seven schools with pre- and post-renovation data. The boxes demonstrate the interquartile range (IQR) of the indoor air quality measure. Whiskers represent closest value within 1.5× the IQR of the indoor air quality measure. Blue dots represent individual values that extend beyond the whisker value. The red line in (**B**) represents the indoor concentration of 1000 ppm recommended by ASHRAE. The red line in (**C**) represents the indoor WHO guidelines of 25 µg/m^3^. All CO values were below the recommended WHO guideline of 7 mg/m^3^ (6.1 ppm at 25 °C). All school daily measurements included were taken between 8:00 a.m. and 4:00 p.m. (**A**) Indoor school daily CO by school renovation status; (**B**) Indoor school daily CO_2_ by school renovation status; (**C**) Indoor school daily PM_2.5_ by school renovation status.

Box plots of school daily indoor air quality data for schools prior to and post-renovation within the seven schools with pre- and post-renovation data, compared to recommended indoor air quality levels, are shown in Figure 1.

The results of the linear mixed models for indoor air quality within the seven schools with pre- and post-renovation data, shown in Table 3, show significant reductions in CO_2_, indoor PM_2.5_, and CO by renovation status after controlling for season. Linear mixed models of indoor air quality within the full sample of 26 pre-renovation and 11 post-renovation schools show similar results (Online Supplement Table S2).

### 3.2. Indoor Air Temperature and Temperature out of Range

**Figure 2:**
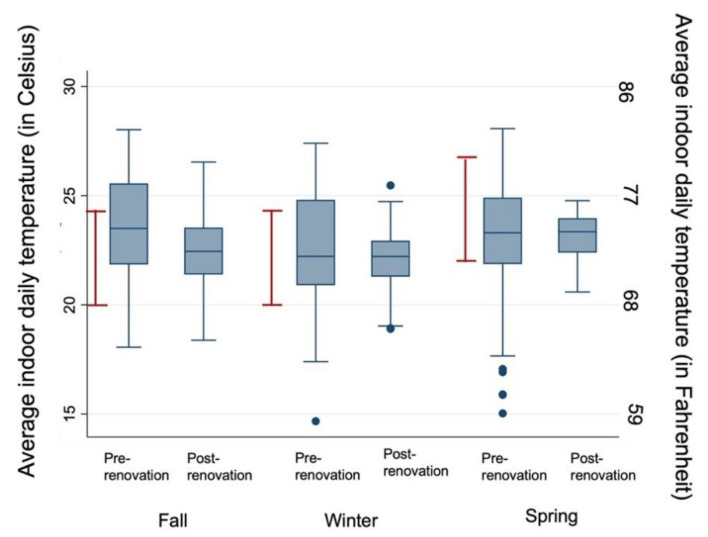
Indoor school daily temperature by school renovation status and season in seven schools with pre- and post-renovation data. The boxes demonstrate the IQR of average indoor daily temperature. Whiskers represent closest value within 1.5× the IQR of the average indoor daily temperature. Blue dots represent individual values that extend beyond the whisker values. Red lines indicate recommended indoor temperature guidelines from ASHRAE: 68–75 °F (20–23.9 °C) in fall/winter and 73–80 °F (22.8–26.7 °C) in the spring/summer. Indoor school daily temperatures were measured between 8:00 a.m. and 4:00 p.m.

Within the seven schools with pre- and post-renovation data, there was decreased temperature variability across seasons following renovation (Table 2, Figure 2). The results of the linear mixed model, in Table 3, show a reduction of proportion of temperature out of range by 21% following renovation after controlling for season. Results of the linear mixed model including all schools in the sample were similar, with a significant reduction in proportion of temperature out of range by 19% (Online Supplement Table S2).

**Figure 3:**
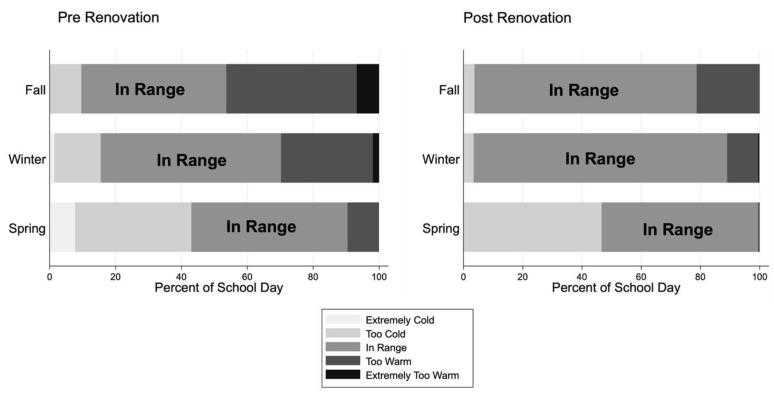
Proportion of indoor temperatures within and outside of range in seven schools with pre- and post-renovation monitoring data. In range indoor air temperatures were determined according to ASHRAE guidelines by season: 68–75 °F (20–23.9 °C) in fall/winter and 73–80 °F (22.8–26.7 °C) in the spring/summer. Temperatures were considered to be too cold or too warm if they were below or above the limit of the ASHRAE recommended range for the season by 4 °C or less. Extreme values were defined as values that were more than 4 °C outside of the ASHRAE recommended range. All indoor temperatures included were taken during the school day, between 8:00 a.m. and 4:00 p.m.

The proportion of indoor air temperatures out of range, by season, prior to and following renovation is shown in Figure 3 (temperature measured every 10 min). Temperatures were more frequently within the recommended range following renovation (72.0%) than prior to renovation (49.4%). Prior to renovation, temperatures were too warm 39.6% of the time in the fall, 27.9% of the time in the winter, and 9.3% of the time in the spring. Additionally prior to renovation, temperatures were extremely too warm 6.8% of the time in the fall, 1.9% of the time in the winter, and 0.2% of the time in the spring. Following renovation, 21.3% of temperatures were too warm in the fall, 10.5% were too warm in the winter, and 0.6% were too warm in the spring. Following renovation, there was improvement in indoor temperature such that no temperatures were extremely too warm in the fall or spring and only 0.5% of temperatures were extremely too warm in the winter.

Prior to renovation, temperatures were too cold 9.6% of the time in the fall, 14.1% of the time in the winter, and 35.3% of the time in the spring. Additionally prior to renovation, temperatures were extremely too cold 1.4% of the time in the winter and 7.8% of the time in the spring. Following renovation, 3.7% of temperatures were too cold in the fall, 3.4% were too cold in the winter, and 46.6% were too cold in the spring. Also, following renovation, there was improvement such that no temperatures were extremely too cold in the fall, winter, or spring. The proportion of indoor air temperatures out of range by season in the complete sample of 29 schools can be seen in Online Supplement Figure S1.

### 3.3. Outdoor Air Quality Measures

Prior to renovation, the two week average outdoor PM_2.5_ was 8.0 µg/m^3^ (IQR: 7.3–14.3 µg/m^3^) and following renovation, it was 6.7 µg/m^3^ (IQR: 5.2–7.5 µg/m^3^). Prior to renovation, median school daily temperature was 20.9 °C (IQR: 18.3–23.2 °C) in the fall, 8.5 °C (IQR: 4.2–14.2 °C) in the winter, and 16.3 °C (IQR: 11.6–23.3 °C) in the spring. Following renovation, median school daily temperature was 20.2 °C (IQR: 14.9–26.7 °C) in the fall, 4.9 °C (IQR: 2.8–8.1 °C) in the winter, and 20.1 °C (IQR: 12.5–25.3 °C) in the spring (Table 2).

The results of the linear mixed models for outdoor air quality (Table 3) showed no difference in outdoor PM_2.5_ by renovation status after controlling for season, and significantly warmer temperatures following renovation compared to prior to renovation.

## 4. Discussion

A school renovation program in an urban, mid-Atlantic school district provided the opportunity to apply a natural experimental design to study the impact of building renovation or replacement on indoor air quality. Prior to renovation, within the seven schools in our sample that had pre- and post-renovation data, nearly one third of indoor CO_2_ measurements exceeded recommended guidelines, and over half of indoor temperature measurements were outside of the recommended range, typically too warm even when temperatures were cool outside. All CO and most PM_2.5_ levels fell within recommended guidelines prior to renovation. Following school renovation, there were improvements in indoor air quality with a significant decrease in CO_2_, indoor PM_2.5_, and CO levels, in addition to an improvement in indoor temperature more frequently within the recommended range for thermal comfort.

The study addresses a recognized research gap by intensively characterizing school indoor air quality with direct assessment at multiple times across different seasons. The unique design provided the opportunity to quantify the impact of an intervention—school renovation or replacement—on indoor air quality. The study was based in an urban school district that serves predominantly low-income, Black children who are disproportionately affected by challenges of aging school infrastructure in urban areas across the United States [31]. The 2011 survey that informed the school renovation program upon which this study was based reported that nearly a quarter of schools were constructed before 1946 (23%), and that the majority (122 out of 145) were rated in poor or worse condition. Only 1 of the 145 schools surveyed was rated in good condition [21]. It is also notable that the schools that were slated to undergo renovation first were those in the worst condition and thus are the schools included in the present study.

Our data shows substantial post-renovation improvement in ventilation, which refers to the exchange of indoor air for clean outdoor air in order to dilute air pollution or contaminants that come from indoor sources and the crowding of people. Notably, among our larger sample of 26 schools that had pre-renovation measurements, more than one-quarter had median CO_2_ values that exceeded the ASHRAE recommended threshold, and this was substantially improved among schools that underwent renovation. Most schools and buildings have HVAC systems with filters on them to maintain good indoor air quality and to provide thermal comfort. In general, the greater number of people in an indoor space, the greater need for ventilation, and thus CO_2_ is used as a surrogate for adequacy of ventilation per occupant. Elevated CO_2_ has been associated with reduced student performance in several states and many countries [19,32,33,34,35], and also associated with an increase in student absences in the US [36]. Marked improvement in ventilation after renovation demonstrates the benefit of investment in school infrastructure with improvement in ventilation, which has the potential to improve standardized test scores and academic performance [34,35], reduce respiratory health effects, and reduce student absences [34]. Of note, in the context of the current SARS-CoV-2 pandemic, there has been increased attention to ventilation characteristics of school buildings. In devising plans to allow children to return to school safely, improving ventilation is one strategy that is being promoted to mitigate airborne viral transmission as well as to improve indoor air quality.

Within the seven schools with pre- and post-renovation data, the majority of baseline average indoor PM_2.5_ concentrations (median 5.9 µg/m^3^, IQR: 3.8–9.5 µg/m^3^) were below the recommended limits for indoor air and consistent with the few other studies that have assessed indoor PM in school environments [37]. PM_2.5_ measured in schools was much lower than PM_2.5_ that have been measured in homes of children in an urban environment in several other research studies [7,38,39,40]. One contributing factor to lower PM_2.5_ levels in schools may be the lack of secondhand smoke (SHS), which is a prominent contributor to household PM_2__.5._ SHS was initially assessed within this study but was discontinued, as it was found to be routinely below the limit of detection (90% of collected samples below the limit of detection). School policies ban smoking on campus and study staff did not observe any evidence of smoking on campus over the study period.

While there were improvements in indoor PM_2.5_ concentrations with renovation, there was no change in concurrently measured outdoor PM_2.5_ concentrations over time, suggesting that improvements in indoor PM_2.5_ were not due to changes in outdoor levels. Despite low baseline PM_2.5_ values, school renovation still resulted in reductions of PM_2.5_ concentrations. This is notable due to evidence suggesting that reduction of PM_2.5,_ even at lower concentrations, may have amplified health benefits, and that there is no evidence of a threshold value, meaning that lower PM is better and there is no safe level of exposure below which there is not the potential for adverse health effects [41,42]. This may be especially relevant for children who have higher respiratory rates and are developing physically (e.g., lung growth, neurocognition) [43].

Thermal comfort was assessed and baseline findings were striking and mirrored the experiences of the study staff regarding the proportion of time that temperature was beyond that in the range for thermal comfort. The extreme values measured were informative. For example, within schools with pre and post renovation data, temperatures were too warm or extremely too warm 46.4% and 29.8% of the time during the fall and winter prior to renovation. There were no extremely too warm values measured post-renovation. It is logical that such thermal conditions may not be conducive to optimal learning. Prior data has shown that indoor temperatures impact student achievement with classroom temperature reduced to the lower end of the comfortable range being associated with increased student work rate, reduced errors, and higher performance scores [20,44]. Higher classroom temperatures have also been shown to be associated with higher daytime attacks of breathlessness [33]. Our findings suggest that renovation greatly improves temperature control with over 20% improvement in the number of days within recommended ranges following school renovation. Perhaps surprisingly, the temperatures were still out of range 28% of the time following renovation, with more temperatures out of range being too cold rather than too warm. This could be due to incomplete benefit of the renovation on the goal of achieving climate control or, alternately, may be due to factors such as the scheduled programming of thermostats that are not responsive to real time weather changes (e.g., heat is turned off in the spring overnight or not turned back on during unexpected cold weather events).

There were several limitations to this study. Given that this study occurred within a natural experiment of an ongoing urban school renovation project and schools were selected based on poor condition, we were unable to randomly assign renovation status to schools within the district or control the extent of renovation performed. The planned renovation timeline for schools extends beyond our study window and thus we sampled more schools pre-renovation than post-renovation. As the renovation project was based in urban schools with low income and minority students, findings may be representative of similar low income communities throughout the country, but are not generalizable to all schools. However, the improvement of indoor school air quality for this vulnerable group of students is particularly important due to known disparities in health and education among low-income communities. Additionally, individual consent from students, teachers, and staff was not a feature of this study. This was intentional in order to reduce burden on the schools and in order to build a working relationship to foster future collaboration. Thus, we were unable to measure individual benefits for these groups. As cost is a significant consideration of school renovation and replacement, cost analysis is also of great interest but is beyond the scope of this study. Measurements of indoor air quality were measured using electrochemical (CO) and NDIR (CO_2_) sensors. These sensors were selected to provide high temporal resolution data enabling an assessment of concentrations during school hours only. However, these sensors can be less accurate and precise than cumulative methods and can be subject to bias based on environmental conditions. Finally, classroom occupancy, which would contribute to ventilation, was not directly measured in this study. As classroom occupancy rates are mandated by school district, occupancy was likely stable prior to and following school renovation.

Due to a history of underinvestment, low income communities have aging school infrastructure that is in significant need of renovation [45]. There is a need to prioritize renovation and replacement of schools within these communities to help address disparities. Our findings show significant improvement in indoor air quality, including CO_2_, indoor PM_2.5_, and CO, as well as an increase in the proportion of indoor temperatures within recommended ranges following school renovation. These findings underscore the need for investment in school renovation to improve building infrastructure and indoor air quality, which may lead to health and academic benefits for students and teachers. Future studies should consider measuring the impact of school renovation and improvements in indoor air quality on student health and performance outcomes.

## Figures and Tables

**Table 1:** Characteristics of schools.

School Statistics	School Variable	No. (% of 29 Total Schools)	No. (% of 7 Schools with Pre- and Post- Renovation Data)
School type	Elementary school only (K-5)	10 (34.5)	1 (14.3)
	Elementary/Middle school (K-8)	15 (51.7)	3 (42.9)
	Middle/High school	1 (3.4)	1 (14.3)
	High school only	3 (10.3)	2 (28.6)
School renovation status	Renovated	9 (31)	5 (71.4)
	Replaced	2 (6.8)	2 (28.6)
Stage of renovation that data was collected	Pre-renovation only	19 (65.5)	0
	Pre-renovation and Post-renovation	7 (24.1)	7 (100)
	Post-renovation only	3 (10.3)	0
		Median (IQR)	Median (IQR)
School characteristics	Facility condition index ^a^	70.6 (59.3–91.75)	76.4 (65.1–96.5)
	Year of construction	1958 (Min: 1910/Max: 1981)	1944 (Min: 1926/Max: 1971)
	% Non-white students ^b^	98.8 (92.7–99.1)	99.1 (85.3–99.8)
	% Free and reduced lunch ^c^	93.7 (89.5–96.1)	91.3 (85.2–94.6)

^a^ Facility Condition Index (FCI) is an industry standard to evaluate building conditions: ≤10% is good condition; 11–30% is average; 31–50% is poor; 51–74% is very poor; ≥75% is a candidate for replacement. FCI was measured in all schools within the Jacobs Report [21]. ^b^ % Non-white students is the percentage of non-white students within the school during the year of entry in the study [29]. ^c^ % Free and Reduced Lunch Meals (FARMS) is the percentage of free and reduced meals provided within the school during the 2014–2015 school year. Baltimore City started offering free and reduced meals to all public school students in June 2015 [30].

**Table 2:** Characteristics of indoor and outdoor air quality and performance outcomes by renovation status in 7 schools with pre- and post-renovation data.

	Pre-Renovation					Post-Renovation				
Indoor Exposures	Season	N1	N2	Median (IQR)	Min, Max	Season	N1	N2	Median (IQR)	Min, Max
School daily CO_2_ (ppm)		7	230	774.0 (626.3–1073.2)	354.1, 2157.1		7	232	542.4 (460.3–679.4)	332.2, 2444
School daily PM_2.5_ (µg/m^3^)		7	204	5.9 (3.8–9.5)	1.0, 25.5		7	252	2.7 (1.4–5.2)	0.7, 17.9
School daily CO (ppm)		7	209	0.4 (0.3–0.6)	0, 1.4		7	179	0.3 (0.1–0.6)	0, 3.1
School daily temperature (°C)	Fall	4	41	23.5 (21.9–25.5)	18.1, 28.0	Fall	6	95	22.5 (21.4–23.5)	18.4, 26.5
	Winter	6	67	22.2 (20.9–24.8)	14.7, 27.4	Winter	6	78	22.2 (21.3–22.9)	18.9, 25.5
	Spring	7	94	23.3 (21.9–24.9)	15.0, 28.0	Spring	6	54	23.4 (22.4–23.9)	20.6, 24.8
**Outdoor Exposures**										
Two week average PM_2.5_ (µg/m^3^)		7	18	8.0 (7.3–14.3)	6.7, 56.1		3	8	6.7 (5.2–7.5)	4.0, 8.5
School daily temperature (°C)	Fall	4	37	20.9 (18.4–23.2)	7.3, 33.6	Fall	4	44	20.2 (14.9–26.7)	1, 37.4
	Winter	5	58	8.5 (4.2–14.2)	−6.1, 23.6	Winter	3	29	4.9 (2.8–8.1)	−4.0, 17.0
	Spring	6	67	16.3 (11.6–23.3)	4.4, 30.7	Spring	4	30	20.1 (12.5–25.3)	8.5, 38.3

N1 = Number of schools monitored. N2 = Number of days monitored (indoor CO, indoor CO_2,_ indoor PM_2.5,_ indoor temperature, outdoor temperature) or number of two week measurements (outdoor PM_2.5_). All school daily measurements were taken between 8:00 a.m. and to 4:00 p.m.

**Table 3:** Linear mixed models comparing indoor air quality within seven schools with pre- and post-renovation monitoring data.

Indoor Exposures	Coefficient	95% CI LB	95% CI UB	*p* Value
Log school daily average PM_2.5_	−0.789	−0.947	−0.630	<0.05
Log school daily average CO	−0.296	−0.472	−0.120	<0.001
Log school daily average CO_2_	−0.345	−0.420	−0.269	<0.001
Proportion of Temperature out of Range	−0.214	−0.318	−0.110	0.435
**Outdoor Exposures**				
Log PM_2.5_	−0.467	−0.860	−0.075	NS
School daily temperature	0.0273	−2.01	2.06	<0.001

Note: PM_2.5_, CO, and CO_2_ have been log transformed. This model controls for season and renovation status. Coefficient values are comparing post-renovation to pre-renovation status, resulting in negative values for the environmental measures that suggest improvement. School daily exposure measurements were taken between 8:00 a.m. and 4:00 p.m. The abbreviation NS stands for not significant.

## Data Availability

The data presented in this study are available on request from the corresponding author. The data are not publicly available due to potential identification.

## Supplementary Materials

The following are available online at https://www.mdpi.com/article/10.3390/ijerph182212149/s1, Figure S1: Proportion of indoor temperatures within and outside of range prior to and post-renovation in 29 schools, Table S1: Characteristics of indoor and outdoor air quality and performance outcomes by renovation status in 29 schools, Table S2: Six linear mixed models showing indoor and outdoor air quality prior to and following school renovation in 29 schools.
