# Supplementary material for: Indoor Air Quality Prior to and Following School Building Renovation in a Mid-Atlantic School District
Source: Int J Environ Res Public Health. 2021 Nov 19;18(22):12149. doi: 10.3390/ijerph182212149 (PMC8624555; doi:10.3390/ijerph182212149)
Supplement: Supplementary file 1 [file ijerph-18-12149-s001.zip › ijerph-1445771-supplementary.pdf]

Online Supplement Figure S1. Proportion of indoor temperatures within and outside of range prior to and post-renovation in 29 schools

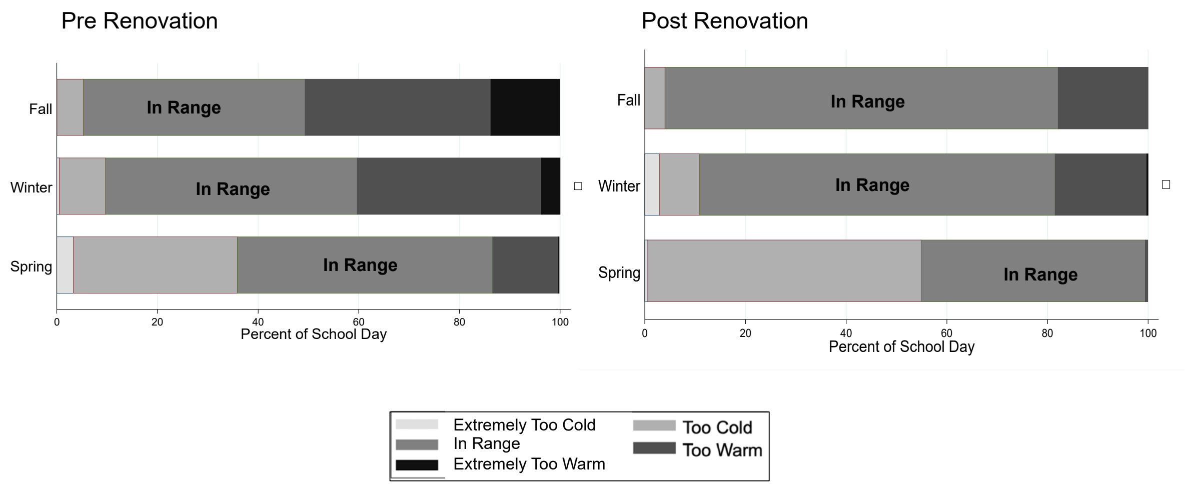

Note: In range indoor air temperatures were determined according to ASHRAE guidelines by season: 68-75 °F (20-23.9 °C) in fall/winter and 73-80 °F (22.8-26.7 °C) in the spring/summer. Temperatures were considered to be too cold or too warm if they were below or above the limit of the ASHRAE recommended range for the season by 4 °C or less. Extreme values were defined as values that were more than 4 °C outside of the ASHRAE recommended range. All indoor temperatures included were taken during the school day, between 8 AM and 4 PM.

Online Supplement Table S1. Characteristics of indoor and outdoor air quality and performance outcomes by renovation status in 29 schools

	Pre-Renovation						Post-Renovation					
Indoor Exposures	Season	N1	N2	Median	IQR	Min, Max	Season	N1	N2	Median	IQR	Min, Max
School daily CO_2 (ppm)		26	880	784.5	629.7-1047.8	354.1, 2157.1		11	324	577.5	478.7-708.9	322.2, 2258.7
School daily $PM_{2.5}$ (µg/m^3)		26	895	4.5	2.3-8.2	0.7, 141.2		11	363	2.9	1.5-6.1	0.7, 34.5
School daily CO (ppm)		25	703	0.4	0.2-0.6	0, 2.1		10	241	0.3	0.1-0.6	0, 2.1
School daily temperature (^{o} C)	Fall	22	259	23.7	21.9-26.3	18.2, 32.1	Fall	9	125	22.2	21.3-23.3	18.4, 25.3
	Winter	24	262	23.3	21.8-24.9	17.8, 29.8	Winter	9	114	22.6	21.4-23.4	15.5, 25.7
	Spring	25	315	23.6	22.2-25.2	15.9, 28.4	Spring	8	90	22.8	21.9-23.8	19.4, 24.8
Outdoor Exposures												
Two week average $PM_{2.5}$ (µg/m^3)		24	62	7.8	6.9-11.2	3.2, 56.1		5	14	7.5	6.2-8.5	4.0, 20.5
School daily temperature (^{o} C)	Fall	20	206	20.9	15.5-25.2	8.3, 33.6	Fall	6	54	21.9	15.7-27.6	1, 37.4
	Winter	21	198	7.1	1.9-11.4	-6.1, 23.6	Winter	5	46	4.5	-0.5-7.7	-9.8, 17.0
	Spring	21	206	17.6	12.8-22.3	4.9, 31.1	Spring	5	49	13.9	8.8-22.5	1.2, 38.3

N1 = Number of schools monitored.
N2 = Number of days monitored (indoor CO, indoor CO_2, indoor $PM_{2.5}$, indoor temperature, outdoor temperature) or number of two week measurements (outdoor $PM_{2.5}$).
All school daily measurements were taken between 8 AM to 4 PM.

Online Supplement Table S2. Six linear mixed models showing indoor and outdoor air quality prior to and following school renovation in 29 schools

**Indoor Exposures**	**Coefficient**	**95% CI LB**	**95% CI UB**	**P Value**
Log school daily average CO_2	-0.323	-0.388	-0.259	<0.001
Log school daily average $PM_{2.5}$	-0.746	-0.825	-0.597	<0.001
Log school daily average CO	-0.256	-0.431	-0.079	<0.05
Proportion of Temperature out of Range	-0.188	-0.274	-0.102	<0.01
**Outdoor Exposures**				
Log $PM_{2.5}$	-0.154	-0.396	0.088	NS
School daily temperature	-1.145	-2.808	0.519	<0.001

Note: $PM_{2.5}$, CO, and CO_2 have been log transformed. This model controls for season and renovation status. Coefficient values are comparing post-renovation to pre-renovation status, resulting in negative values for the environmental measures. School daily exposure measurements were taken between 8 AM and 4 PM.
